# Validation of the Peruvian Spanish Version of the Stress and Anxiety to Viral Epidemics-6 Scale to Measure Viral Anxiety of Medical Students During COVID-19

**DOI:** 10.3389/fpsyt.2022.876379

**Published:** 2022-05-31

**Authors:** André Lapeyre-Rivera, Nair Javier-Murillo, Francisco Perea-Flórez, Bryan Gamonal, Víctor Velásquez-Rimachi, Carlos Alva-Díaz, Oli Ahmed, Seockhoon Chung

**Affiliations:** ^1^Facultad de Medicina, Universidad Nacional Mayor de San Marcos, Lima, Peru; ^2^Sociedad Científica de San Fernando, Universidad Nacional Mayor de San Marcos, Lima, Peru; ^3^Red de Eficacia Clínica y Sanitaria (REDECS), Lima, Peru; ^4^Facultad de Medicina Humana, Universidad de Piura, Lima, Peru; ^5^Sociedad Científica de la Universidad de Piura, Universidad de Piura, Lima, Peru; ^6^Grupo de Investigación en Neurociencia, Efectividad Clínica y Salud Pública, Universidad Científica del Sur, Lima, Peru; ^7^Departamento de Medicina y Oficina de Apoyo a la Docencia e Investigación (OADI), Servicio de Neurología, Hospital Daniel Alcides Carrión, Callao, Peru; ^8^Department of Psychology, University of Chittagong, Chattogram, Bangladesh; ^9^National Centre for Epidemiology and Population Health, Australian National University, Canberra, ACT, Australia; ^10^Department of Psychiatry, Asan Medical Center, University of Ulsan College of Medicine, Seoul, South Korea

**Keywords:** medical students, COVID-19, SAVE-6, anxiety, stress, Peru

## Abstract

**Introduction:**

The COVID-19 pandemic has created academic problems for Peruvian medical students leading to anxiety and depression. Hence, validated scales, such as the Stress and Anxiety to Viral Epidemics-6 items (SAVE-6), are required to identify and propose interventions to improve mental health. We aimed to perform a psychometric validation of the Peruvian version of SAVE-6 on medical students during the COVID-19 pandemic in Lima, Peru, in 2022.

**Methods:**

A total of 260 medical students at National University of San Marcos (UNMSM) participated in an online survey in January 2022. We collected sociodemographic characteristics and classified psychiatric symptoms using SAVE-6, the Generalized Anxiety Disorder-7 items (GAD-7) scale, and the Patient Health Questionnaire-9 items (PHQ-9). We performed confirmatory and parallel factor analysis to examine the validity of the Peruvian Spanish version of SAVE-6.

**Results:**

We explored the reliability and validity of SAVE-6 and SAVE-6 after excluding item 5, since factor loading of item 5 is too low. Both scales showed good internal consistencies (Cronbach's α = 0.780 and.82 and McDonald's Ω = 0.792 and.829, respectively). Furthermore, SAVE-6 after excluding item 5 showed good convergent validity with GAD-7 (*r* = 0.224, *p* <.001) and PHQ-9 (*r* = 0.217, *p* <.001). Consequently, instead of the full SAVE-6, SAVE-6 excluding item 5 proved to be reliable and valid enough to assess the anxiety of Peruvian medical students during the pandemic.

**Conclusion:**

The Peruvian Spanish SAVE-6 scale excluding item 5, rather than the full SAVE-6, can be applied to measure viral anxiety of medical students in Peru with good validity and reliability.

## Introduction

The COVID-19 pandemic has had a considerable impact worldwide. As the World Health Organization (WHO) stated, deaths due to COVID-19 have exceeded five million, of which more than two million have been in the Americas (1). According to the data reviewed on March 15th 2022, ~200,000 deaths have been reported in Peru^1^, making it the country with the highest mortality rate (650.80 deaths per 100,000 inhabitants) and the third highest fatality rate (6.9%) in the world (2).

As part of the contingency plan for the pandemic, Peruvian universities established physical distancing measures that included only online classes, which meant no hospital rotations for medical students. This has been a mental challenge for students and may have frustrated their personal expectations. A systematic review and meta-analysis reported an increased prevalence of depression (39%) and anxiety (36%) in university students during the pandemic (3). Specifically, in medical students in China, the presence of COVID-19-related psychological distress was evident in ~27% of participants and 11% showed an acute stress reaction (4). Another study involving Chinese medical students during the initial phase of the pandemic reported that 0.9% of students showed severe anxiety symptoms, 2.7% had moderate symptoms, and 21.3% had mild symptoms (5). In Peru, an observational study on university students from different regions found that 51.2% of them showed medium-to-moderate levels of anxiety and 45% showed medium-to-high levels of depression (6). Additionally, a study conducted on first-year medical students in Lima, Peru revealed that 75.4% of participants manifested some degree of anxiety during COVID-19 (7).

Evidence suggests that the COVID-19 pandemic has a negative global impact on the mental health of students. College students often face stressful events such as a difficult study program, difficult assignments and projects, financial problems, and uncertainty about their future, and such challenges must be faced emotionally. This translates into higher rates of stress and anxiety compared to those in the general population (8). Evidently, during the pandemic period, mental health problems, such as anxiety (9), depression (10), and post-traumatic stress disorder (11), have increased. In a study by Son et al. (12) on university students, 71% of the participants had increased stress and anxiety levels owing to the COVID-19 pandemic. Additionally, studies show that the prevalence of depression, anxiety, and/or suicidal thoughts has assumed alarming proportions among college students (13). In turn, studies indicate that medical students are particularly vulnerable to poor mental well-being and psychological distress compared to the general population. In a research conducted by Saddik et al. (14), anxiety levels and the effects of online education on anxiety levels differed between medical students and the general university population.

Medical students must maintain optimal mental health through specific measures to ensure the continuity of their education and their subsequent successful professional practice, which involves promotion, prevention, and intervention in people's health (15, 16). Medical schooling is inherently a challenging and stressful academic experience that can make medical students vulnerable to depression, anxiety, and burnout. Studies investigating the mental health of practicing physicians have shown that stress that begins in medical school tends to continue throughout the years of medical practice (15). After medical school, a physician also often lends himself to a chronically stressful lifestyle (16). Owing to the COVID-19 pandemic, medical students are unprepared for situations of uncertainty in clinical practice, which can generate anxiety and stress and impact mental wellbeing (17). Therefore, simple questionnaires are needed to enable appropriate screening of these problems, as timely detection of anxiety in medical students is critical to proposing specific interventions to maintain their mental health and ensure proper development of their academic activities and future professional work.

There are scales to measure these problems, such as the COVID-19 Anxiety Scale (CAS-7) (18), the Fear of COVID-19 Scale (FCV-19S) (19), the Coronavirus Anxiety Scale (CAS) (20), and the Stress and Anxiety to Viral Epidemics-6 Items (SAVE-6) scale (21). CAS-7 (18) and FCV-19S (19) include items pertaining to worry, increased heartbeat, or repetitive thoughts, and CAS (20) includes items of physiological arousal symptoms associated with clinically elevated fear and anxiety. In our unpublished work on comparing these scales in the general population (22), CAS was the most discriminating and difficult, SAVE-6 was the least discriminating, and CAS-7 and FCV-19S were moderately discriminating. In this study, we attempted to screen medical students who needed psychological support for their anxiety regarding the pandemic in clinical clerkship rather than screening them for mental health impairments, and we considered that SAVE-6 was appropriate for the study objective.

The SAVE-6 is a self-reported questionnaire, in which each item is graded according to a Likert scale, receiving a value of 0 (never) to 4 (always), with a total score between 0 and 24 (21). It was developed to measure individual levels of anxiety regarding the pandemic among the general population derived from one of two factors (factor I—anxiety about the epidemic) of the SAVE-9 scale, which was developed for assessing work-related stress and anxiety specifically in response to the COVID-19 epidemic (23). SAVE-6 has already been validated in the general populations in South Korea (21), Lebanon (24), Bangladesh (25), and the United States (26). It was also applied with good reliability and validity to special populations, such as public-sector workers (27), cancer patients (28), medical students (29), and healthcare workers (30). In a study conducted on medical students in South Korea (29), where the psychometric properties and convergent validity of SAVE-6 were explored, the single-factor structural model of SAVE-6 was found to have good internal consistency (Cronbach's α = 0.756) and good convergent validity with the Generalized Anxiety Disorder-7 items (GAD-7) scale, CAS, and Work and Social Adjustment Scale (WSAS). Moreover, through receiver operating characteristic analysis, the appropriate cutoff score was determined as 15 points in accordance with at least a mild degree of generalized anxiety.

The present study was developed during the so-called third wave that referred to the rapid increase in positive cases of COVID-19 between December 2021 and March 2022^2^. With the vaccination process, which has slowed down^2^, and the constant political changes in Public Health (7 Ministers of Health since the first reported case of COVID-19 in Peru), there is no good expectation in the short and medium terms. Since the situation is not favorable in Peru, the necessary preventive measures in Public Health for new outbreaks or different pandemics are not being taken, and obtaining a reliable and valid instrument to measure anxiety due to COVID-19 among medical students in Peru is crucial, the validation of SAVE-6 could be an attractive alternative because standardized health indicators will be available for international comparative studies (31). Therefore, we aimed to perform a psychometric validation of the Peruvian version of SAVE-6 on medical students during the COVID-19 pandemic in Lima, Peru, in 2022.

## Methods

### Participants and Procedure

For this psychometric validation study, we calculated a sample of 281 students (with a type 1 error of 5% and an estimated proportion of 50%) for a total finite population of 1,050 medical students enrolled at National University of San Marcos (UNMSM) during the 2021–22 period. We collected data through an online survey in Google Forms in January 2022. During this time, we received results from 260 students, which represents an estimated sample for a type 1 error of 5.3%. The survey was sent through institutional emails provided by the UNMSM medical school. In Peru, medical school is divided into 7 years. In general, the curriculum is divided into pre-medical (first year), pre-clinical (second and third years), clinical (fourth to sixth years), and medical internship (seventh year) where the students gain practical experience in hospitals. In this study, students who provided consent and provided complete information were eligible to participate. The survey form was developed according to the Checklist for Reporting Results of Internet e-Surveys (CHERRIES) guidelines (32), and investigators checked the usability and technical functionality of the survey form before implementation. The study protocol was approved by the Research Ethics Committee of the Faculty of Medicine of UNMSM (application #0165).

### Sociodemographic Characteristics

We collected the following data: age, sex, and grade (number of years the student is studying at the time of the survey and can vary from 1 to 7 years).

### Measures

#### Stress and Anxiety to Viral Epidemics-6 Items Scale

The SAVE-6 scale is a self-reported rating scale, which was developed for assessing one's pandemic-related anxiety (21). It was derived from the original SAVE-9 scale, which was developed to measure healthcare workers' occupational stress and anxiety response to the COVID-19 epidemic (23). SAVE-9 was clustered around two factors: Factor I—“Anxiety about the epidemic” (items 1, 2, 3, 4, 5, and 8, namely SAVE-6) (21) and Factor II— “Work-related stress associated with the epidemic” (items 6, 7, and 9, namely SAVE-3) (33). In this study, we applied the SAVE-6 scale, which can be applied to the general population, rather than SAVE-9, which was developed for healthcare workers. While medical students can play roles similar to healthcare workers, they do not actually work as healthcare workers. Item 9 of SAVE-9 (“Do you think that your colleagues would have more work to do due to your absence from a possible quarantine and might blame you?”) was not appropriate for application to medical students who do not work and are not replaced by other medical students. Additionally, items 6 and 7 can be confusing to medical students (29); hence, we determined SAVE-6 to be more applicable to medical students.

The 6 items of SAVE-6 are rated on a Likert scale ranging from 0 (never) to 4 (always). A higher total score reflects higher levels of anxiety. In this study, we translated the SAVE-6 scale using a translation and back-translation method. The translation team comprised a bilingual Peruvian translator expert in linguistics, who oversaw both the direct and reverse translation process. Together with this expert, the researchers oversaw semantic verification of the Spanish version of the SAVE-6 questionnaire (https://www.save-viralepidemic.net) to adapt it to the Peruvian context. To check the understanding of the adaptation, we conducted a pilot test with 30 medical students who were not included in our last sample.

#### Generalized Anxiety Disorder-7 Items

The GAD-7 scale is a self-reported scale for measuring the severity of general anxiety (34). The 7 items of GAD-7 are rated on a 4-point Likert scale ranging from 0 (not at all) to 3 (nearly every day). A higher total score means higher levels of general anxiety. The Spanish version of GAD-7 (from Spain) was previously applied in Peru on medical students (7), which was validated in Spain among the general population (35). In this study, we applied the Spanish version of GAD-7 (35), and Cronbach's α in this sample was 0.736.

#### Patient Health Questionnaire-9 Items

The PHQ-9 is a self-reported rating scale for measuring the severity of depression (36). The 9 items of PHQ-9 are rated on a 4-point Likert scale ranging from 0 (not at all) to 3 (nearly every day). A high total score means severe levels of depression. In this study, we applied the Peruvian Spanish version of PHQ-9 (37) validated among medical students, and Cronbach's α in this sample was 0.883.

### Statistical Analysis

We explored the construct validity and reliability of the Peruvian Spanish version of SAVE-6. Normality was checked based on skewness and kurtosis values of each item within the range ± 2 (38). To check the sampling adequacy and data suitability for factor analyses, the Kaiser–Meyer–Olkin (KMO) value and Bartlett's test of sphericity were examined. Confirmatory factor analysis (CFA) with the diagonally weighted least squares method was conducted to check the factor structure of the Peruvian Spanish version of SAVE-6. A satisfactory model fit for the factor structure was defined by a standardized root-mean-square residual (SRMR) value ≤.05, root-mean-square-error of approximation (RMSEA) value ≤.10, and comparative fit index (CFI) and Tucker–Lewis index (TLI) values ≥.90 (39, 40). Multi-group CFA was conducted to examine whether the Peruvian Spanish version of SAVE-6 can measure the pandemic-related anxiety of medical students in the same way between sexes, among those with depression (PHQ-9 ≥ 10), or those with anxiety (GAD-7 ≥ 10). Internal consistency reliability was tested based on Cronbach's α and McDonald's Ω. Convergent validity of the Peruvian Spanish SAVE-6 with pre-existing GAD-7 and PHQ-9 scales were tested by Pearson's correlation coefficients. Psychometric properties were also assessed by conducting the item response theory (IRT) approach [graded response model (GRM)] and the Rasch model. Before running the GRM, IRT assumptions [unidimensionality (Loevinger's *H* coefficient), local dependence [*p*-values of *G*^2^: adjusted for false discovery rate, FDR], and monotonicity [number of significant violations and *Crit* value]) were examined. Furthermore, item fits (assessed through S-χ^2^ and its *p*-values [adjusted for FDR]) were assessed. In GRM, there are two parameters for items: in - slope/ discrimination parameter (α) and threshold/ difficulty parameters (*b*). For both parameters in GRM, local dependence and item fits were estimated using the R package *mirt* version 1.34. Unidimensionality and monotonicity were estimated through R package *mokkoen* version 3.0.6. In addition, IRT reliability was also calculated. In the Rasch model, infit mean square (infit MnSQ), outfit MnSQ, item difficulty, item and person separation index, and item and person reliability were estimated. Differential item functioning (DIF) bias across age, either having depression (PHQ-9 ≥ 10) or having anxiety (GAD-7 ≥ 10), estimated Mantel–Haenszel χ^2^. SPSS version 21.0 (IBM, Armonk, NY), AMOS version 27 (IBM), JASP version 0.14.1.0 software (JASP Team, Amsterdam, Netherlands), Rasch analysis, and DIF were run through jMetrik version 4.1.1 (Psychomeasurement Systems, Charlottesville, VA), and RStudio (RStudio, Boston, MA) was used for statistical analysis.

## Results

### Population Characteristics

Among the 260 UNMSM medical students who participated in this study, 50.8% (132/260) were male; thus, the male/female ratio was 1.03. The participants' median age was 22 years (Q1: 20; Q3: 23.5). Most students (61.5%) had completed the first 3 years of medical school (163/260) (Table 1). The participants' mean rating scale results were 13.3 ± 4.1 (range: 4–24), 9.8 ± 3.7 (range: 0–20), and 11.8 ± 5.7 (range: 0–27) for SAVE-6, GAD-7, and PHQ-9, respectively (Table 1).

**Table 1:** Demographic characteristics of participants.

**Variables**	**Mean ±SD, *n* (%)**
**Sex (male) (*N* = 260)**	132 (50.8%)
**Grade (*N* = 260)**	
1st year	48 (18.4%)
2nd year	52 (20%)
3rd year	63 (24.2%)
4th year	34 (13.1%)
5th year	33 (12.7%)
6th year	21 (8.1%)
7th year	9 (3.5%)
**Rating scale scores**	
Stress and Anxiety to Viral Epidemics-6 items (SAVE-6)	13.3 ± 4.1 (4~24)
Generalized Anxiety Disorder-7 items (GAD-7)	9.8 ± 3.7 (0~20)
Patient Health Questionnaire-9 items (PHQ-9)	11.8 ± 5.7 (0~27)

*N, Total number of medical students; n, sample*.

*SD, standard deviation*.

### Factor Structure and Psychometric Properties of SAVE-6

#### Peruvian Spanish Version of SAVE-6

The distribution of six items of the Peruvian Spanish version of SAVE-6 was within the normal limit based on the skewness and kurtosis for an acceptable limit range of ±2 (Table 2). Sample adequacy and data suitability for conducting factor analysis were checked using the KMO measure of 0.80 and Bartlett's test of sphericity (*p* <.001). CFA had a good model fit [χ2 (df, *p*-value) = 13.290 (9, 0.150), CFI = 0.993, TLI = 0.988, RMSEA = 0.043, SRMR = 0.051, Table 3) for the single-factor model of SAVE-6. However, the factor loading value of item 5 was too low (0.251, Table 2).

**Table 2:** Factor structure of the Peruvian Spanish versions of SAVE-6 and SAVE-6 excluding item 5.

**Items**	**Response scale**					**Descriptive**				**SAVE-6**			**SAVE-6 excluding item 5**		
	**0**	**1**	**2**	**3**	**4**	**M**	**SD**	**Skewness**	**Kurtosis**	**CITC**	**CID**	**Factor loading**	**CITC**	**CID**	**Factor loading**
**Item 1**	2.7	15.8	40.4	30.8	10.4	2.30	0.948	−0.094	−0.306	0.565	0.738	0.620	0.541	0.811	0.603
**Item 2**	2.3	21.5	40.4	24.6	11.2	2.21	0.980	0.143	−0.524	0.624	0.723	0.737	0.742	0.752	0.745
**Item 3**	2.7	23.5	39.6	24.2	10.0	2.15	0.982	0.155	−0.516	0.710	0.700	0.848	0.667	0.775	0.855
**Item 4**	9.6	33.5	33.1	18.5	5.4	1.77	1.034	0.251	−0.495	0.536	0.745	0.642	0.583	0.801	0.653
**Item 5**	10.0	28.5	34.2	19.2	8.1	1.87	1.090	0.172	−0.585	0.228	0.824	0.251	-	-	-
**Item 6**	0.4	4.2	22.7	38.5	34.2	3.02	0.881	−0.550	−0.329	0.573	0.738	0.636	0.568	0.804	0.626

*0 = never, 1 = rarely, 2 = sometimes, 4 = often, 5 = always*.

*M, mean; SD, standard deviation; CITC, corrected item-total correlation; CID, Cronbach's α if item deleted; CI, confidence interval; SAVE-6, Stress and Anxiety to Viral Epidemics-6 items*.

**Table 3:** Scale-level psychometric properties of the Peruvian Spanish versions of SAVE-6 and SAVE-6 excluding item 5.

**Scales**	**SAVE-6**	**SAVE-6 excluding item 5**	**Suggested cutoff**
**Psychometric properties**	**Scores**		
Floor effect	0	0	15%
Ceiling effect	0.8	1.5	15%
Mean inter-item correlation	0.382	0.484	Between 0.15 and 0.50
Cronbach's α	0.780	0.824	≥0.7
McDonald's Ω	0.792	0.829	≥0.7
Split-half reliability (odd-even)	0.793	0.789	≥0.7
Standard error of measurement	1.92	1.55	Smaller than SD (5.25)/2
Loevinger's *H* coefficients	0.412	0.536	-
*Rho* coefficient	0.788	0.864	≥0.7
IRT reliability	0.863	0.837	≥0.7
Item separation index	7.639	8.583	≥2
Person separation index	2.018	2.322	≥2
Item reliability	0.983	0.987	≥0.7
Person reliability	0.803	0.844	≥0.7
**Statistics from exploratory factor analysis**			
KMO measure of sample adequacy	0.80	0.80	0.50
Bartlett's test of sphericity	489.1955, *p* < 0.001	464.258, *p* < 0.001	Significant
Determinant	0.148	0.164	
Eigenvalue	3.03	2.95	1 or above
Variance	50.45	59.01	
RSMR	0.060	0.050	
TLI	0.888	0.900	
**Output from parallel analysis**			
Reduced eigenvalue	2.472	2.400	1 or above
95th percentile of random reduced eigenvalue	0.325	0.279	
**Model fits of confirmatory factor analysis**			
*χ^2^* (df, *p*-value)	13.290 (9, 0.150)	7.183 (5, 0.207)	Non-significant
CFI	0.993	0.996	>0.95
TLI	0.988	0.992	>0.95
RMSEA	0.043	0.041	<0.08
SRMR	0.051	0.044	<0.08

*SAVE-6, Stress and Anxiety to Viral Epidemics-6 items; IRT, item response theory; KMO, Kaiser–Meyer–Olkin; RSMR, root-mean-square residual; TLI, Tucker–Lewis index; CFI, comparative fit index; RMSEA, root-mean-square-error of approximation; SRMR, standardized root-mean-square residual*.

#### Peruvian Spanish Version of SAVE-6 Excluding Item 5

Since the factor loading value of item 5 in the Peruvian Spanish version of SAVE-6 was too low, we checked the psychometric properties of the scale excluding item 5. The KMO measure of 0.80 and Bartlett's test of sphericity (*p* < 0.001) showed the sample was adequate and data were suitable for conducting factor analysis for SAVE-6 excluding item 5. CFA had improved good model fits (χ2 [df, *p*-value] = 7.183 (5, 0.207), CFI = 0.996, TLI = 0.992, RMSEA = 0.041, SRMR = 0.044, Table 3) for the single-factor structure model of SAVE-6 excluding item 5. Factor loading values of the items ranged between 0.603 and 0.855, Cronbach's α between 0.752 and 0.811, and corrected item-total correlations between 0.541 and 0.742 (Table 2). Multi-group CFA results (Supplementary Table S1) showed strict invariance of SAVE-6 excluding item 5 across sexes, in those with depression (PHQ-9 ≥ 10), and in those with anxiety (GAD-7 ≥ 10).

### Reliability of SAVE-6 and SAVE-6 Without Item 5 and Evidence-Based on Relations to Other Variables

#### Peruvian Spanish Version of SAVE-6

The Peruvian Spanish version of SAVE-6 showed good internal consistency (Cronbach's α = 0.780, McDonald's Ω = 0.792) and good convergent validity based on Pearson's correlation coefficient with GAD-7 (*r* = 0.252, *p* <.001) and PHQ-9 (*r* = 0.242, *p* <.001) scores. The SAVE-6 score was significantly higher among participants with anxiety [GAD-7 ≥ 10, *t*(258) = 14.719, *p* <.001] and depression [PHQ-9 ≥ 10, *t*(258) = 4.978, *p* <.001].

#### Peruvian Spanish Version of SAVE-6 Excluding Item 5

The SAVE-6 showed good internal consistency (Cronbach's α = 0.820, McDonald's Ω = 0.829) and good convergent validity based on Pearson's correlation coefficient with GAD-7 (*r* = 0.224, *p* < 0.001) and PHQ-9 (*r* = 0.217, *p* <.001) scores when item 5 was excluded. The total score was significantly higher among participants with anxiety [GAD-7 ≥ 10, *t*(258) = 13.144, *p* < 0.001] and depression [PHQ-9 ≥ 10, *t*(258) = 4.615, *p* < 0.001].

### Graded Response Model

#### Peruvian Spanish Version of SAVE-6

**Figure 1:**
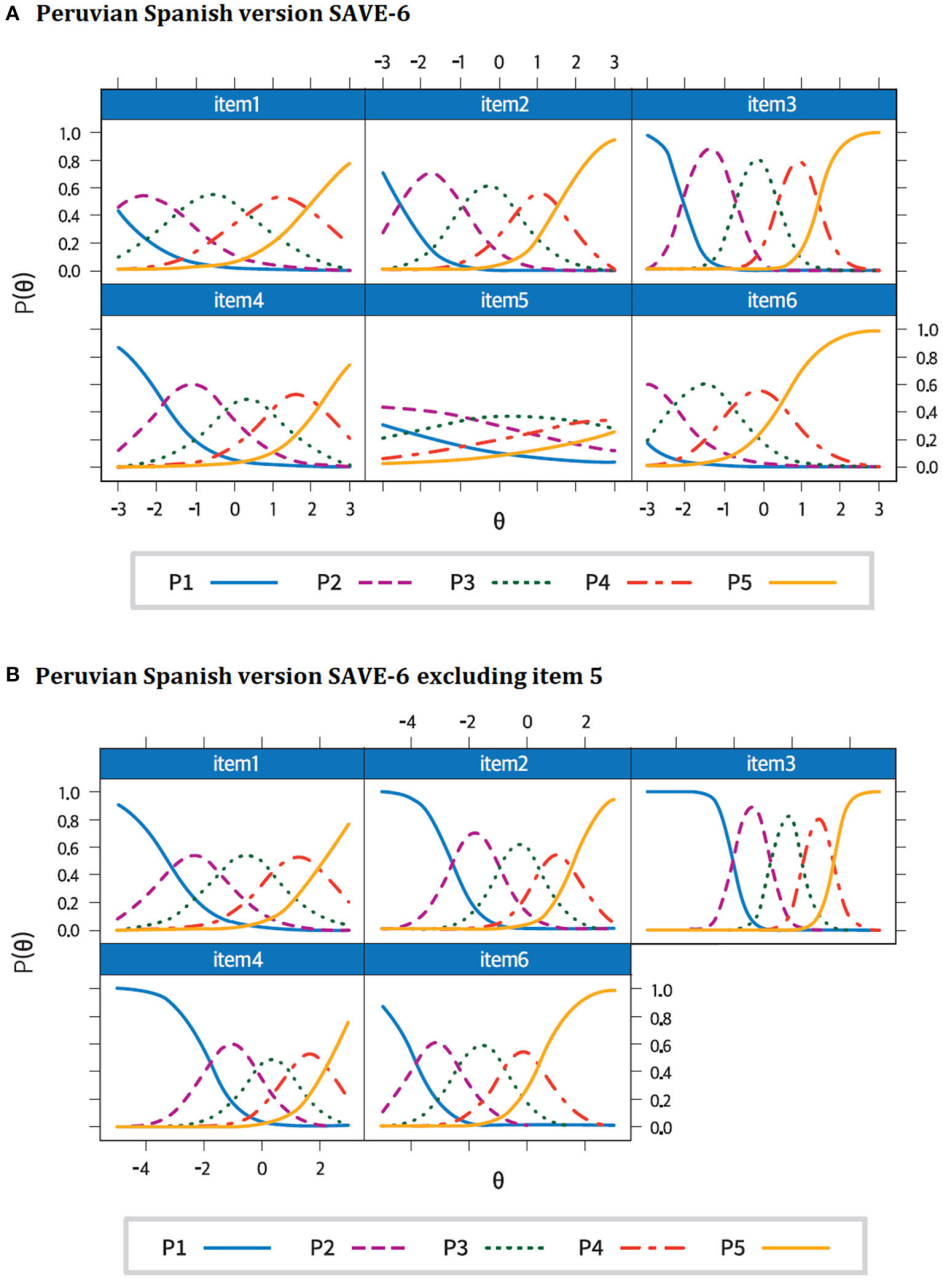
Threshold characteristics curves of the Peruvian Spanish versions of SAVE-6 **(A)** and SAVE-6 excluding item 5 **(B)**. SAVE-6, Stress and Anxiety to Viral Epidemics-6 items.

Information about IRT assumptions is presented in Table 3 and Supplementary Table S2. Loevinger's *H* coefficient (0.412; Table 3) suggested that the Peruvian version of SAVE-6 was moderately unidimensional. Non-significant *p*-values of *G*^2^ (Supplementary Table S2) suggested the absence of local dependency between items. The absence of significant violation and the low value of the *Crit* statistic (<40) for all items indicated that the monotonicity assumption was valid. Results regarding Loevinger's *H* coefficient, *G*^2^, and monotonicity suggested the suitability to run an IRT model. Supplementary Table S3 presents the item fit statistics of the Peruvian version of SAVE-6. After controlling the FDR, the *p*-values of S-χ^2^ indicated that all items fit the scale well, which suggested that all the items belong to the scale. The slope/discrimination parameters (α) ranged between 0.472 and 4.031 (mean = 1.912) (Supplementary Table S3). Item 5 had a low slope, item 1 had a high slope, and the rest of the items had a very high slope. All items except item 5 provided reasonable information and were more efficient in discriminating among individuals assessed through the Peruvian version of SAVE-6. The threshold coefficients (b) in Supplementary Table S3 suggested that a higher latent trait or theta was required to endorse Likert-type response options from “often” to “always” in all items except item 6. Threshold characteristics curves (Figure 1A) showed that curves for item 5 were very flat. This suggested the non-suitability of item 5.

#### Peruvian Spanish Version of SAVE-6 Excluding Item 5

Loevinger's *H* coefficient (0.536; Table 3) suggested that the Peruvian version of SAVE-6 was highly unidimensional, even when item 5 was excluded. Non-significant *p*-values of *G*^2^ (Supplementary Table S2), the absence of significant violation, and the low value of the *Crit* statistic (<40) for all items (Supplementary Table S2) suggested that the local dependence and monotonicity assumption was valid. Supplementary Table S3 presents the item fit statistics of the Peruvian version of SAVE-6 excluding item 5. After controlling the FDR, the *p*-values of S-χ^2^ indicated that all items fit the scale well, which suggested that all the items belong to the scale. The slope/discrimination parameters (α) ranged between 1.327 and 4.173 (mean = 2.218) (Supplementary Table S3). Item 1 had a moderate slope, whereas the rest of the items had a very high slope. All items provided reasonable information and were more efficient in discriminating among individuals assessed through the Peruvian version of SAVE-6 excluding item 5. Item 1 provided the least information and item 3 provided the most information about the latent trait. The threshold coefficients (b) in Supplementary Table S3 suggested that a higher latent trait or theta was required to endorse Likert-type response options: from “often” to “always” in all items except Item 6. Figure 1B presents the threshold characteristics curves of the Peruvian version of SAVE-6. Supplementary Figure S1 shows the scale information curves of the Peruvian Spanish version of SAVE-6 and SAVE-6 excluding item 5. Both scales were good for assessing the latent trait between −2.5 and 2.0 theta levels. However, there were several peaks in both curves that might be due to polytomous data.

### The Rasch Model

#### Peruvian Spanish Version of SAVE-6

Supplementary Table S4 presents the Rasch model outputs of the Peruvian version of SAVE-6. Infit and mean squares of all the items were between the recommended range (0.50–1.50) except item 5. Items 6 and 4 had the lowest and highest item difficulty, respectively.

#### Peruvian Spanish Version of SAVE-6 Excluding Item 5

Supplementary Table S4 presents the Rasch model outputs of the Peruvian version of SAVE-6 when item 5 was excluded. Infit and mean squares of all the items were within the recommended range (0.50–1.50). Items 6 and 4 had the lowest and highest item difficulty, respectively. DIF results (Supplementary Table S5) showed an absence of DIF bias in items across sexes, in those with depression (PHQ-9 ≥ 10), and in those with anxiety (GAD-7 ≥ 10).

## Discussion

In this study, we aimed to explore the psychometric properties of the Peruvian Spanish version of SAVE-6 among medical students in Peru. We observed that SAVE-6 was a reliable and valid rating scale, which could assess medical school students' pandemic-related anxiety. The CFA showed a good model fit for the single-factor model of SAVE-6. Multi-group CFA showed that the Peruvian Spanish version of SAVE-6 can measure medical students' pandemic-related anxiety in the same way across sexes and in students with depression (PHQ-9 ≥ 10) or anxiety (GAD-7 ≥ 10). It also showed good internal consistency and convergent validity with other anxiety scales, such as GAD-7 and PHQ-9.

In this study, we assessed the efficiency of the Peruvian Spanish version of SAVE-6 *via* GRM, a modern test theory model. Similar to the classic test theory, the unidimensionality test (Loevinger's *H* coefficient) confirmed the single-factor structure of both the Peruvian Spanish versions of SAVE-6 and SAVE-6 excluding item 5. The item fit values of SAVE-6 excluding item 5 confirmed that all items were included on the scale. Similar to the results of the factor analyses, item 5 of SAVE-6 had low slope parameters, and the threshold characteristic curves of this item were flat. However, all items of SAVE-6 excluding item 5 were sufficient to discriminate high scores from low scores. Both versions efficiently assessed the latent trait between −2.5 and 2.0 theta levels. The Rasch analysis infit and outfit MnSqs showed similar results of factor analysis and GRM, and item 5 had higher infit and outfit MnSqs than the recommended value (0.5–1.5). These outputs suggested the unsuitability of this item. In both versions, item 6 was the least difficult, and item 4 was the most difficult. Both versions had the acceptable item and person separation indices and reliability. DIF results suggested consistent results with multi-group CFA. There was an absence of DIF bias in items of the Peruvian version of SAVE-6 across sexes, in those with depression, and in those with anxiety.

However, we observed that the factor loading value of item 5 (“Are you worried that others might avoid you even after the infection risk has been minimized?”) in the Peruvian Spanish version of SAVE-6 was too low (0.251) among medical students in Peru. The reasons may be as follows. First, item 5 may not be useful to assess one's pandemic-related anxiety. Originally, SAVE-9 was clustered around two factors: pandemic-related anxiety (items 1, 2, 3, 4, 5, and 8) and work-related stress (items 6, 7, and 9). However, in Russia (41) and Germany (42), item 5 was not clustered around a pandemic-related anxiety subscale, but rather a work-related subscale, among a sample of healthcare workers. Therefore, we can speculate that item 5 can be useful in measuring the work-related stress of healthcare workers rather than anxiety in response to a viral epidemic. Second, a sampling of medical students, who may play a similar role to healthcare workers, may influence the results. Similar results were observed in a sample of healthcare workers in Spain (30). In this study, the factor loading value of item 5 was low (0.38). Thus, the validity of the single-factor model of the Peruvian Spanish version of SAVE-6 needs to be checked in the general population. Third, cultural differences might influence the results. The clusters observed in studies conducted in Japan (43) and Turkey (44) were in parallel with the Korean study but differed in countries such as Russia and Germany. SAVE-6 showed a good fit for a single-factor model among the general populations of Korea (21), Lebanon (24), and the US (26). Further studies are needed to explore the differences in SAVE-6 clustering in other countries.

This study has some limitations. First, it was not possible to arrive at the previously calculated sample. However, the gap between the sample obtained and the one calculated is small (5.3% vs. 5%). Therefore, despite not being able to reach our target population sample, the results obtained from the psychometric validation process are reliable. In any case, we suggest corroborating these in various student populations. Additionally, there was heterogeneity in the percentages of students in each grade due to the poor response of students in the higher grades. Hence, this sample may not be representative of our population. This may have been due to the unavailability of Internet access or to the greater academic load that students in the final grades of medical school bear. However, we re-sent the survey over 4 weeks to reach as many participants as possible. Second, due to the current pandemic situation, face-to-face surveys have not been possible, and surveys had to be conducted online. Anonymous online surveys are likely to induce response biases. Third, the GAD-7 version applied in this study was not validated among medical students in Peru in Peruvian Spanish. We applied the European Spanish version of GAD-7, which might influence the results. Finally, the low factor loading value of item 5 might have come from the sampling issue, although we already compared the characteristics of item 5 among European countries. In the future, when SAVE-6 is applied to other samples, researchers should consider whether item 5 will be included or not.

In conclusion, the Peruvian Spanish version of SAVE-6 excluding item 5, rather than the full SAVE-6, can be applied to measure pandemic-related anxiety of medical students in Peru with good validity and reliability. In the current COVID-19 pandemic, this scale would be helpful in assessing the psychological problems of medical students.

## Data Availability Statement

The raw data supporting the conclusions of this article will be made available by the authors, without undue reservation.

## Ethics Statement

Ethical approval for all procedures and analyses conducted for the current manuscript was provided by the Research Ethics Committee of the Faculty of Medicine of UNMSM (application 0165). Electronic informed consent was obtained from all individual participants.

## Author Contributions

AL-R, NJ-M, FP-F, and BG contributed to the acquisition of data. SC, OA, VV-R, and CA-D contributed to the analysis and interpretation of data. All authors agreed to be accountable for all aspects of the work to ensure that questions related to the accuracy or integrity of any part of the work were appropriately investigated and resolved. All authors contributed substantially to the conception and design of the study and to the article and approved the submitted version.

## Conflict of Interest

The authors declare that the research was conducted in the absence of any commercial or financial relationships that could be construed as a potential conflict of interest.

## Publisher's Note

All claims expressed in this article are solely those of the authors and do not necessarily represent those of their affiliated organizations, or those of the publisher, the editors and the reviewers. Any product that may be evaluated in this article, or claim that may be made by its manufacturer, is not guaranteed or endorsed by the publisher.

## Footnotes

^1^https://covid19.minsa.gob.pe/sala_situacional.asp (accessed March 23, 2022).

^2^https://www.minsa.gob.pe/reunis/data/vacunas-covid19.asp (accessed March 23, 2022).
