# Supplementary material for: Validation of the Peruvian Spanish Version of the Stress and Anxiety to Viral Epidemics-6 Scale to Measure Viral Anxiety of Medical Students During COVID-19
Source: Front Psychiatry. 2022 May 31;13:876379. doi: 10.3389/fpsyt.2022.876379 (PMC9193401; doi:10.3389/fpsyt.2022.876379)
Supplement: Supplementary file 1 [file Data_Sheet_1.docx]

**Supplementary Table S1. Measurement invariance of SAVE-6 excluding item 5 across sexes, in those with depression, and those with anxiety**

| **Model** | **χ^2^** | **Df** | **Δ χ^2^** | **Δdf** | **p** | **CFI** | **ΔCFI** | **RMSEA** | **ΔRMSEA** |
| --- | --- | --- | --- | --- | --- | --- | --- | --- | --- |
| **Multi-group variable: Sex (male vs. female)** | | | | | | | | | |
| Configural | 8.395 | 10 |  |  |  | 1.000 |  | .000 |  |
| Metric | 12.111 | 14 | 3.716 | 4 | 0.446 | 1.000 | 0 | .000 | 0 |
| Scalar | 14.222 | 18 | 2.111 | 4 | 0.715 | 1.000 | 0 | .000 | 0 |
| Strict | 18.095 | 23 | 3.873 | 5 | 0.568 | 1.000 | 0 | .000 | 0 |
| **Multi-group variable: generalized anxiety (GAD-7 ≥ 10 vs. GAD-7 < 10)** | | | | | | | | | |
| Configural | 8.793 | 10 |  |  |  | 1.000 |  | .000 |  |
| Metric | 11.585 | 14 | 2.792 | 4 | 0.593 | 1.000 | 0 | .000 | 0 |
| Scalar | 15.466 | 18 | 3.881 | 4 | 0.422 | 1.000 | 0 | .000 | 0 |
| Strict | 20.684 | 23 | 5.218 | 5 | 0.389 | 1.000 | 0 | .000 | 0 |
| **Multi-group variable: generalized anxiety (PHQ-9 ≥ 10 vs. PHQ-9 < 10)** | | | | | | | | | |
| Configural | 8.375 | 10 |  |  |  | 1.000 |  | .000 |  |
| Metric | 8.734 | 14 | 0.359 | 4 | 0.986 | 1.000 | 0 | .000 | 0 |
| Scalar | 14.176 | 18 | 5.442 | 4 | 0.245 | 1.000 | 0 | .000 | 0 |
| Strict | 17.24 | 23 | 3.064 | 5 | 0.690 | 1.000 | 0 | .000 | 0 |

CFI, comparative fit index; RMSEA, root-mean-square-error of approximation; GAD-7, Generalized Anxiety Disorder-7 items; PHQ-9, Patient Health Questionnaire-9 items

**Supplementary Table S2. Loevinger’s H coefficient, monotonicity, and G^2^ p-values of items of the Peruvian Spanish versions of SAVE-6 and SAVE-6 excluding item 5**

|  | *H* coefficients | Monotonicity | | | | Local dependence G^2^ p values | | | | |
| --- | --- | --- | --- | --- | --- | --- | --- | --- | --- | --- |
|  |  | #ac | #vi | #zsig | *Crit* | Item1 | Item2 | Item3 | Item4 | Item5 |
| **(A) SAVE-6** | | | | | | | | | | |
| Item1 | .44 | 18 | 0 | 0 | 0 |  |  |  |  |  |
| Item2 | .47 | 18 | 0 | 0 | 0 | .206 |  |  |  |  |
| Item3 | .52 | 13 | 0 | 0 | 0 | .581 | .670 |  |  |  |
| Item4 | .42 | 11 | 0 | 0 | 0 | .794 | .581 | .574 |  |  |
| Item5 | .19 | 24 | 2 | 0 | 35 | .581 | .559 | .075 | .581 |  |
| Item6 | .45 | 14 | 1 | 0 | 9 | .670 | .581 | .581 | .670 | .382 |
| **(B) SAVE-6 excluding item 5** | | | | | | | | | | |
| Item1 | .54 | 18 | 0 | 0 | 0 |  |  |  |  |  |
| Item2 | .61 | 16 | 0 | 0 | 0 | .213 |  |  |  |  |
| Item3 | .67 | 13 | 0 | 0 | 0 | .694 | .694 |  |  |  |
| Item4 | .67 | 20 | 0 | 0 | 0 | .802 | .694 | .694 |  |  |
| Item6 | .57 | 15 | 1 | 0 | 17 | .694 | .694 | .694 | .694 |  |
| ac = active comparison, vi = violation, zsig = significant violation  SAVE-6, Stress and Anxiety to Viral Epidemics-6 items.  Notes: p-values adjusted for false discovery rate (FDR) | | | | | | | | | | |

**Supplementary Table S3. Item fit and slope and threshold parameters of the Peruvian Spanish versions of SAVE-6 and SAVE-6 excluding item 5**

| **Items** | **Item fits** | | | **Slope parameter (a)** | **Threshold parameter (b)** | | | |
| --- | --- | --- | --- | --- | --- | --- | --- | --- |
|  | **S-χ^2^** | **df** | **p value** |  | **b_1_** | **b_2_** | **b_3_** | **b_4_** |
| **(A) SAVE-6** | | | | | | | | |
| Item 1 | 19.56 | 25 | .805 | 1.359 | -3.191 | -1.436 | .342 | 2.069 |
| Item 2 | 17.22 | 19 | .805 | 2.080 | -2.579 | -.940 | .440 | 1.626 |
| Item 3 | 12.77 | 18 | .805 | 4.031 | -2.048 | -.697 | .395 | 1.438 |
| Item 4 | 32.47 | 27 | .805 | 1.705 | -1.865 | -.263 | .986 | 2.340 |
| Item 5 | 38.44 | 37 | .805 | .472 | -4.813 | -1.003 | 2.200 | 5.353 |
| Item 6 | 19.872 | 20 | .805 | 1.822 | -3.846 | -2.317 | -.813 | .540 |
| **(B) SAVE-6 excluding item 5** | | | | | | | | |
| Item 1 | 14.918 | 19 | .828 | 1.327 | -3.242 | -1.456 | .347 | 2.099 |
| Item 2 | 11.529 | 17 | .828 | 2.071 | -2.584 | -.941 | .441 | 1.632 |
| Item 3 | 11.310 | 12 | .828 | 4.173 | -2.035 | -.695 | .392 | 1.435 |
| Item 4 | 18.366 | 20 | .828 | 1.726 | -1.854 | -.264 | .980 | 2.325 |
| Item 6 | 16.687 | 16 | .828 | 1.793 | -3.885 | -2.334 | -.818 | .545 |
| SAVE-6, Stress and Anxiety to Viral Epidemics-6 items.  Notes: p-values adjusted for false discovery rate (FDR) | | | | | | | | |

**Supplementary Table S4. Item statistics of the Peruvian Spanish versions of SAVE-6 and SAVE-6 excluding item 5 using Rasch model**

| **Items** | **Infit MnSq** | **Outfit MnSq** | **Difficulty** |
| --- | --- | --- | --- |
| **(A) SAVE-6** | | | |
| Item1 | .85 | .85 | -.12 |
| Item2 | .78 | .78 | .03 |
| Item3 | .62 | .63 | .12 |
| Item4 | 1.05 | 1.05 | .74 |
| Item5 | 1.85 | 1.86 | .57 |
| Item6 | .83 | .79 | -1.33 |
| **(B) SAVE-6 excluding item 5** | | | |
| Item1 | 1.17 | 1.17 | -.01 |
| Item2 | .88 | .88 | .19 |
| Item3 | .70 | .70 | .30 |
| Item4 | 1.21 | 1.20 | 1.09 |
| Item6 | .84 | 1.01 | -1.56 |

SAVE-6, Stress and Anxiety to Viral Epidemics-6 items; MnSq, mean square.

**Supplementary Table S5. Differential item functioning bias across sexes, in those with depression, and in those with anxiety in items of the Peruvian Spanish version of SAVE-6 excluding item 5**

| **Items** | **Sex** | | **GAD-7 ≥ 10** | | **PHQ-9 ≥ 10** | |
| --- | --- | --- | --- | --- | --- | --- |
|  | **MH χ^2^** | **p** | **MH χ^2^** | **p** | **MH χ^2^** | **p** |
| **Item1** | .26 | .61 | 2.26 | .13 | 2.86 | .09 |
| **Item2** | .84 | .36 | .29 | .59 | .47 | .49 |
| **Item3** | 2.37 | .12 | .37 | .54 | .77 | .38 |
| **Item4** | 1.59 | .21 | 2.46 | .12 | 1.44 | .23 |
| **Item6** | .82 | .37 | 1.22 | .27 | 1.88 | .17 |

SAVE-6, Stress and Anxiety to Viral Epidemics-6 items; GAD-7, Generalized Anxiety Disorder-7 items; PHQ-9, Patient Health Questionnaire-9 items.


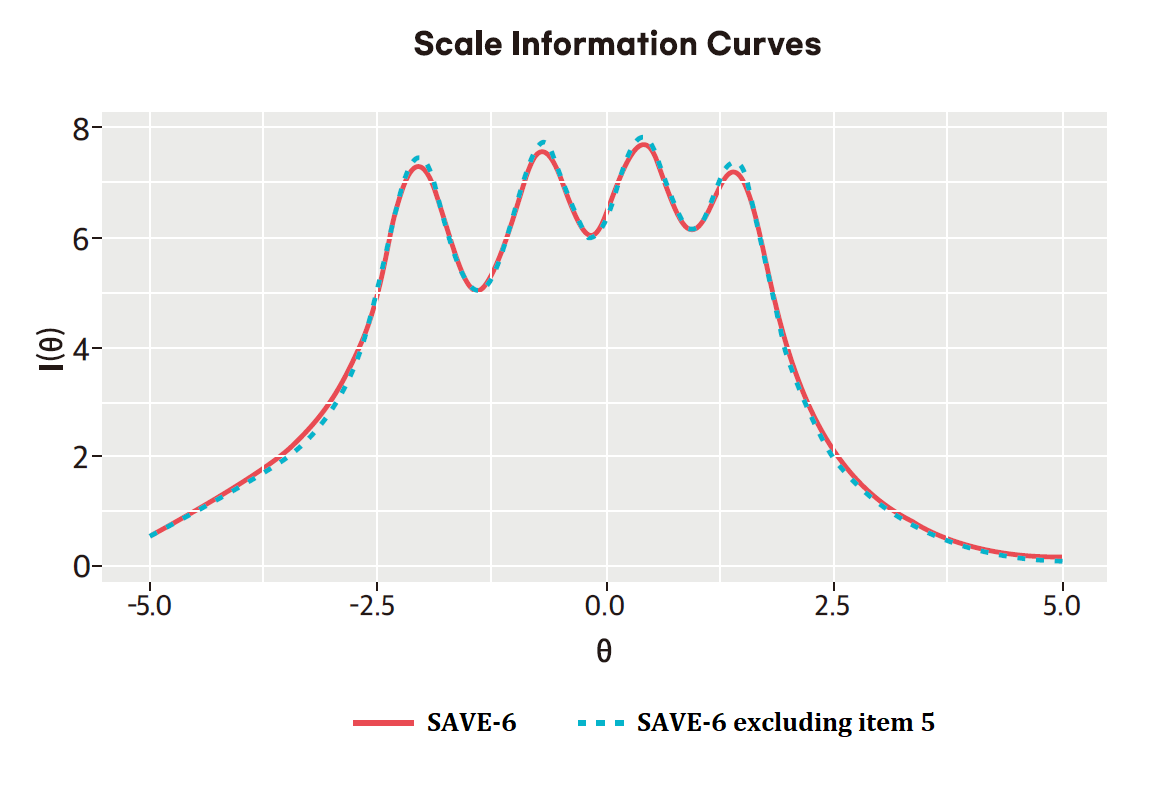


**Supplementary Figure S1. Scale information curves of the Peruvian Spanish versions of SAVE-6 and SAVE-6 excluding item 5. SAVE-6, Stress and Anxiety to Viral Epidemics-6 items.**
